# Myeloablative conditioning in cord blood transplantation for acute myeloid leukemia patients is efficacious only until age 55

**DOI:** 10.1038/s41409-025-02508-2

**Published:** 2025-01-21

**Authors:** Shinichiro Oshima, Yasuyuki Arai, Tadakazu Kondo, Shingo Yano, Shigeki Hirabayashi, Naoyuki Uchida, Makoto Onizuka, Shigesaburo Miyakoshi, Masatsugu Tanaka, Satoshi Takahashi, Masayuki Hayashi, Toshiro Kawakita, Yasufumi Uehara, Shuichi Ota, Toru Izumi, Masashi Sawa, Tetsuya Nishida, Yuta Katayama, Koji Nagafuji, Koji Kato, Tatsuo Ichinohe, Yoshiko Atsuta, Masamitsu Yanada

**Affiliations:** 1 Department of Hematology, Kyoto University Hospital, Kyoto, Japan; 2 Division of Clinical Oncology Hematology, Department of Internal Medicine, The Jikei University School of Medicine, Tokyo, Japan; 3 Division of Precision Medicine, Kyushu University Graduate School of Medical Science, Fukuoka, Japan; 4 Department of Hematology, Federation of National Public Service Personnel Mutual Aid Associations Toranomon Hospitalsociations Toranomon Hospital, Tokyo, Japan; 5 Department of Hematology and Oncology, Tokai University School of Medicine, Tokyo, Japan; 6 Department of Hematology, Tokyo Metropolitan Geriatric Hospital, Tokyo, Japan; 7 Department of Hematology, Kanagawa Cancer Center, Kanagawa, Japan; 8 Department of Hematology/Oncology, Clinical Precision Research Platform, The Institute of Medical Science, The University of Tokyo, Tokyo, Japan; 9 Department of Hematology, Hamanomachi Hospital, Fukuoka, Japan; 10 Department of Hematology, NHO Kumamoto Medical Center, Kumamoto, Japan; 11 Department of Hematology, Kitakyushu City Hospital Organization, Kitakyushu Municipal Medical Center, Fukuoka, Japan; 12 Department of Hematology, Sapporo Hokuyu Hospital, Hokkaido, Japan; 13 Department of Hematology, National Hospital Organization Sendai Medical Center, Miyagi, Japan; 14 Department of Hematology and Oncology, Anjo Kosei Hospital, Aichi, Japan; 15 Department of Hematology, Japanese Red Cross Aichi Medical Center Nagoya Daiichi Hospital, Aichi, Japan; 16 Department of Hematology, Hiroshima Red Cross Hospital & Atomic-bomb Survivors Hospital, Hiroshima, Japan; 17 Division of Hematology and Oncology, Department of Medicine, Kurume University Hospital, Fukuoka, Japan; 18 Central Japan Cord Blood Bank, Aichi, Japan; 19 Department of Hematology and Oncology, Research Institute for Radiation Biology and Medicine, Hiroshima University, Hiroshima, Japan; 20 Japanese Data Center for Hematopoietic Cell Transplantation / Department of Registry Science for Transplant and Cellular Therapy, Aichi Medical University School of Medicine, Aichi, Japan; 21 Department of Hematology and Oncology, Nagoya City University East Medical Center, Aichi, Japan

**Keywords:** Risk factors, Haematopoietic cell growth factors

## Abstract

Umbilical cord blood transplantation (CBT) is accepted as an effective treatment for acute myeloid leukemia (AML), and reduced-intensity conditioning (RIC), rather than myeloablative conditioning (MAC) regimens allowed elderly patients to be treated safely. However, appropriate intensities of conditioning regimens are still unclear, especially for middle-aged patients. To compare outcomes after RIC and MAC regimens, we analyzed AML patients aged 16 years or older in the Japanese registry database, who underwent single cord unit CBT between 2010-2019. Median ages of the RIC group (*n* = 1353) and the MAC group (*n* = 2101) were 59 and 51 years (*P* < 0.001), respectively. 5-year overall survival (OS) after MAC was superior to that of RIC (38.3% vs 27.7%, *P* < 0.001) with lower incidence of relapse (33.9% vs 37.4%, *P* = 0.029) and better neutrophil engraftment (84.7% vs 75.9%, *P* < 0.001). Detailed subgroup analysis revealed that age at transplantation is the most important factor affecting 5-year OS in RIC and MAC. This analysis identified a threshold of 55 years, beyond which the superiority of MAC disappeared, irrespective of other factors such as disease status or performance status. In conclusion, RIC may be preferable for patients aged 56 or older in CBT for AML due to higher potential toxicities.

## Introduction

Cord blood transplantation (CBT) is a highly useful, alternative treatment for acute myeloid leukemia (AML) in cases in which a human leukocyte antigen (HLA)-matched donor is unavailable [1–3]. Since the first CBT performed in 1988, the number of CBTs has grown exponentially [4, 5]. Japanese cohort data revealed that CBT accounted for one-third of all allogeneic transplants for AML [6].

Another notable trend is the steadily increasing number of older patients, with over half of recipients being over 50 years old [6]. This has resulted from development of reduced-intensity conditioning (RIC), which enables this treatment to be used for older patients [7–13]. RIC is generally less toxic than myeloablative conditioning (MAC) and can be beneficial to reduce transplant-related mortality (TRM), but it can increase the incidence of relapse after transplant due to insufficient intensity needed to irradicate all residual tumor cells. The balance of risks of TRM and relapse after CBT is challenging in the context of RIC vs MAC, and factors influencing the choice of conditioning intensity among AML patients are still unclear [5, 14–18]. Regarding the optimal age threshold, Ringdén et al. [19] found that RIC was preferable for patients over 50 years of age in terms of NRM in unrelated donor transplants for AML. Similarly, Shimoni et al. [20] demonstrated that leukemia-free survival was comparable after MAC and RIC in HLA-matched transplants for patients aged 50–55 years (36% vs. 40%, *p* = 0.32), while RIC offered a significant advantage in patients over 55 years (28% vs. 20%, *p* = 0.02). Recently, Akahoshi et al. [21] proposed the Risk Index for Conditioning Intensity in the Elderly (RICE) score, which incorporates advanced age (≥60 years), HCT-CI index (≥2), and the use of CBT to predict the risk of NRM associated with MAC versus RIC. Their findings suggested that RIC may reduce the risk of NRM in older CBT recipients (HR, 0.57; 95% CI, 0.43–0.77; *p* < 0.001). While age appears to play a pivotal role in the choice between RIC and MAC, there is no definitive age threshold, particularly for CBT. Thus, further real-world, data-driven studies comparing RIC and MAC in CBT, especially in middle-aged and elderly patients, are necessary clarify the appropriate population, particularly by age, for which RIC or MAC should be recommended.

Therefore, we hypothesize that by comparing outcome of RIC and MAC in single unit cord blood transplantation using large-scale Japanese registry data analysis, the prognostic factors that could help to determine the intensity of conditioning regimens especially among middle age groups could be identified.

## Patients and methods

### Data collection

Transplant data were obtained from the Transplant Registry Unified Management Program of the Japanese Society for Transplantation and Cellular Therapy (JSTCT)/Japanese Data Center for Hematopoietic Cell Transplantation (JDCHCT). Adult patients (age ≥16 years) with AML who underwent their first umbilical cord blood transplantation (CBT) between 2010 and 2019 in Japan were included. Patients without survival data or without HLA mismatch information were excluded. This study was planned by the Adult AML Working Group of the JSTCT. All patients provided written informed consent for research. The study was conducted according to the Declaration of Helsinki and approved by the Institutional Review Board of Kyoto University, and the Data Management Committees of JSTCT and JDCHCT.

### Conditioning regimens and GVHD prophylaxis

Criteria for RIC and MAC were determined based on Center for International Blood and Marrow Transplant Research (CIBMTR) consensus [22]. RIC was total-body irradiation (TBI) < 5 Gy (single) or TBI < 8 Gy (fraction), poBU dose ≦ 8 mg/kg, ivBU dose ≦6.4 mg/kg, MEL dose ≦140 mg/m^2^. MAC was mainly categorized as TBI-based MAC or high-dose chemotherapy-based MAC. TBI-based MAC included TBI > 6 Gy (single) or TBI > 10 -12 Gy (fraction) with/without cyclophosphamide (120 mg/kg), cytarabine (6–12 g/m^2^), etoposide (30–60 mg/kg), busulfan or fludarabine. High-dose chemotherapy-based MAC included busulfan (16 mg/kg orally or 12.8 mg/kg intravenously) with fludarabine (120–180 mg/m^2^; Flu/Bu4) with/without melphalan (140–180 mg/m^2^), cyclophosphamide, a low dose of TBI, or busulfan (16 mg/kg po, 12.8 mg/kg iv) with cyclophosphamide (120 mg/kg). Acute GVHD prophylactic protocols were registered as 6 groups; Cyclosporin-A (CyA) + Methotrexate (MTX), CyA without MTX, Tacrolimus (Tac) + MTX, TAC without MTX, None, Others.

### Study endpoints and definitions

The primary endpoint was overall survival (OS), which is defined as the time from CBT to the last date of follow-up or any cause of death. Secondary endpoints were relapse/progression-free survival (PFS), defined as AML relapse/progression and death as events, GVHD/relapse-free survival (GRFS), defined as AML relapse, acute/chronic GVHD and death as events, and cumulative incidence of relapse and TRM, defined as death without evidence of AML progression or relapse. Neutrophil engraftment was defined as the first day of neutrophil count ≥0.5 × 10^9^ /L for 3 consecutive days, without evidence of autologous reconstitution or graft rejection within the first 100 days of CBT. Acute and chronic GVHD were diagnosed and graded using standard criteria [23, 24]. Eastern cooperative oncology group performance status scale (ECOG PS) at transplantation was evaluated according to ECOG criteria [25]. Hematopoietic cell transplantation-specific comorbidity index (HCT-CI) was determined according to the Seattle scale [26]. HLA matching was assessed using serological data for the HLA-A, -B and -DR loci [27]. HLA mismatch was defined in the graft-versus-host disease (GVHD) direction when recipient alleles or antigens were not shared by the donor and was defined in the host-versus-graft direction when donor alleles were not shared by the recipient. In this study, complete remission (CR) referred to morphological CR, which was defined as <5% blasts in cellular marrow with recovery of >1000/μL neutrophils, >100,000/μL platelets, and no requirement of red blood cell transfusion, nor evidence of extramedullary leukemia [28].

### Statistical analysis

Patients were divided into two groups based on the conditioning regimen: RIC and MAC. The patient characteristics were evaluated using the chi-square test for categorical variables and the Wilcoxon rank-sum test for continuous variables. Probabilities of OS, PFS and GRFS were evaluated using the Kaplan-Meier method and compared among groups with the Cox proportional-hazard model. Probabilities of relapse, TRM, neutrophil or platelet engraftment and acute or chronic GVHD were evaluated based on cumulative incidence methods to account for competing risks and compared among groups with the Fine-Gray proportional-hazard model [29]. Competing events were death without progression/relapse for progression/relapse, death without engraftment for engraftment, progression/relapse for TRM, and death without acute or chronic GVHD for acute and chronic GVHD. Chronic GVHD was assessed for patients who survived for at least 100 days after transplantation. The following covariates were considered in the multivariate analyses; intensity of conditioning regimens (RIC vs MAC), sex, HCT-CI, ECOG PS, disease status at the time of transplantation, HLA mismatch, donor-sex mismatch, GVHD prophylaxis. Subgroup analyses of age heterogeneity among RIC vs MAC for OS were performed using Cox models, and results are shown using forest plots [30]. All statistical tests were two-sided and *p* values of <0.05 were considered statistically significant. All analyses were performed with R (version 4.3.2).

## Results

### Patient characteristics

Patients and disease characteristics are described in Table 1. In total, 3454 patients with a median age of 54 years (range, 16–80) underwent single cord unit CBT. Among them, 1997 (57.8%) were male and 1457 (42.2%) were female. De novo AML was diagnosed in 3121 (90.4%), whereas 333 (9.6%) were secondary AML. MAC was administered to 2101(60.8%) patients and 1353 (39.2%) patients received RIC. The median age was higher in the RIC group (59 years) compared to the MAC group (51 years). The MAC cohort comprised better ECOG PS scores (PS 0–1; 86.6% vs 73.1%, PS 2–4; 13.3% vs 26.6%) with better HCT-CI scores (HCT-CI 0–2; 77.4% vs 73.5%, HCT-CI 3-: 21.3% vs 24.0%). Tacrolimus-based GVHD prophylaxis regimens were used more frequently than Cyclosporin-A, with their usage being lower in the MAC group compared to the RIC group (CyA with MTX; 18.5% vs 10.1%, CyA without MTX; 2.5% vs 7.4%, Tac with MTX; 42.4% vs 44.7%, Tac without MTX; 35.3% vs 36.2%). In vivo T cell depletion, including the use of ATG, was performed in 141 patients, with 53 patients (2.5%) in the MAC group and 88 patients (6.5%) in the RIC group. The median time from diagnosis to CBT was 184 days with 13.9% receiving CBT within 3 months in MAC, whereas CBT was 264 days with 9.5% receiving it within 3 months in RIC.

**Table 1:** Patient characteristic receiving MRC or RIC regimens.

Variables	Group	MAC *N* = 2101 (60.8%)	RIC *N* = 1353(39.2%)	*p*-value	
Age at CBT, year	Median (range)	51 (16–80)	59 (16–79)	<0.001	***
	Over 50	1090 (51.9%)	1008 (74.5%)		
	50 or under	1011(48.1%)	345(25.5%)	<0.001	***
Sex	Male	1199 (57.1%)	798 (59.0%)		
	Female	902 (42.9%)	555(41.0%)	0.28	
ECOG PS score	0–1	1820 (86.6%)	989 (73.1%)		
	2–4	279 (13.3%)	360 (26.6%)	<0.001	***
HCT-CI score	0–2	1627 (77.4%)	995 (73.5%)		
	3-	448 (21.3%)	325 (24.0%)	<0.001	***
Disease status	CR1	603 (28.7%)	388 (28.7%)		
	CR2	236 (11.2%)	136 (10.1%)		
	CR3-	25 (1.2%)	39 (2.9%)		
	non-CR	1237 (58.9%)	789 (58.3%)	0.003	**
Disease risk	High	1262 (60.1%)	828 (61.2%)		
	Low	839 (39.9%)	524 (38.7%)	0.51	
Disease type	de novo	1921 (91.4%)	1200 (88.7%)		
	Secondary	180 (8.6%)	153 (11.3%)	0.009	**
Time from diagnosis to CBT					
	<3 months	292 (13.9%)	129 (9.5%)		
	3–6 months	665 (31.7%)	306 (22.6%)		
	>6months	1143 (54.4%)	918 (67.8%)	<0.001	***
HLA mismatch	0/6	87 (4.1%)	93 (6.9%)		
	1/6	450 (21.4%)	309 (22.8%)		
	2/6	1549 (73.7%)	942 (69.6%)		
	3/6 or more	15 (0.7%)	9 (0.7%)	0.002	**
GVHD prophylaxis	CyA-based	442 (21.0%)	237 (17.5%)		
	Tac-based	1633 (77.7%)	1095 (80.9%)	0.04	*
Years of CBT	2010–2015	1214 (57.8%)	883 (65.3%)		
	2016–2019	887 (42.2%)	470 (34.7%)	<0.001	***
Median total cell dose (10^7^ cells/kg)		0.27	0.27	0.73	
Median CD34+ cell dose (10^5^cells/kg)		0.9	0.87	0.13	
Median follow-up of survivors (year)		3.24	3.27	0.4	

*PS* performance status, *HCT-CI* hematopoietic cell transplantation-specific comorbidity index, *HSCT* hematopoietic stem cell transplantation, *CR* complete remission, *CBT* cord blood transplantation, *CyA* cyclosporine A, *Tac* Tacrolimus.

*p < 0.05; **p < 0.01; ***p < 0.001.

The most frequently used MAC regimens were CY/TBI + CA (23.1%), followed by FLU/BU4 + MEL (20.9%) and FLU/BU4 + low-dose TBI (13.5%) (Table 2). The most frequently used RIC regimens were FLU/MEL + low-dose TBI (36.6%), followed by FLU/CY + low-dose TBI (12.3%) and Flu/BU2 + low-dose TBI (10.2%) (Table 3).

**Table 2:** Distribution of detailed regimens among MAC patients.

Category	Regimens	*N* = 2101
TBI-based	CY/TBI + CA	485 (23.1%)
	CY/TBI	243 (11.6%)
	CY/TBI + ETP	22 (1.0%)
	others	70 (3.3%)
Chemo-based	FLU/BU4 + MEL	440 (20.9%)
	FLU/BU4+lowTBI	283 (13.5%)
	FLU/BU4 + MEL + CA	197 (9.4%)
	FLU/BU4+lowTBI+CA	101 (4.8%)
	BU/CY	64 (3.0%)
	FLU/Bu4	63 (3.0%)
	FLU/BU4 + CY	39 (1.9%)
	others	94 (4.5%)

*CY* cyclophosphamide, *TBI* total body irradiation, *CA* cytarabine, *ETP* etoposide, *FLU* fludarabine, *BU* busulfan, *MEL* melphalan, *lowTBI* low dose TBI.

**Table 3:** Distribution of detailed regimens among RIC patients.

Category	Regimens	*N* = 1353
FLU/BU based	FLU/BU2 + lowTBI	138 (10.2%)
	FLU/BU2/MEL	88 (6.5%)
	FLU/MEL + BU + CA	27 (2.0%)
	FLU/BU2	23 (1.7%)
	Others	43 (3.2%)
FLU/CY based	FLU/CY + lowTBI	166 (12.3%)
	FLU/CY	26 (1.9%)
	Others	19 (1.4%)
FLU/MEL based	FLU/MEL + lowTBI	495 (36.6%)
	FLU/MEL	114 (8.4%)
	FLU/MEL + lowTBI + CA	69 (5.1%)
	FLU/MEL + CA	44 (3.3%)
	Others	8 (0.6%)
FLU + others	FLU + others	39 (2.9%)
Others		54 (4.0%)

*FLU* fludarabine, *BU* busulfan, *lowTBI* low dose total body irradiation, *MEL* melphalan, *CA* cytarabine, *CY* cyclophosphamide.

### Post-transplant outcomes

**Fig. 1:**
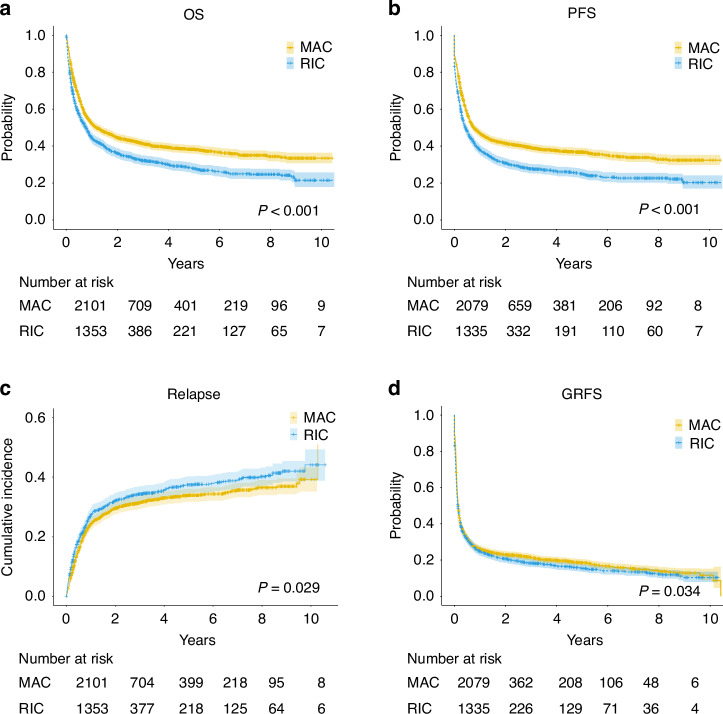
Post-transplant outcomes stratified according to MAC vs RIC regimens among all patients. **a** Overall survival (OS), **b** Progression-free survival (PFS), **c** Cumulative incidence of relapse, and **d** Cumulative incidence of GVHD-free, relapse-free survival are shown. *p* values were calculated using the log-rank test (**a**, **b**) and Fine and Gray’s tests (**c**, **d**). *p  <  0.05.

Patients who received MAC regimens showed significantly higher 5-year OS (38.3% vs 27.7%, *P* < 0.001) and PFS (36.7% vs 24.8%, *P* < 0.001) than those who received RIC regimens (Fig. 1a, b). The 5-year and cumulative incidence of relapse was lower in MAC than RIC (33.9% vs 37.4%, *P* = 0.029) (Fig. 1c). The 5-year GRFS was higher in MAC and RIC regimens (18.5% vs 15.3%, *P* = 0.034) (Fig. 1d).

**Fig. 2:**
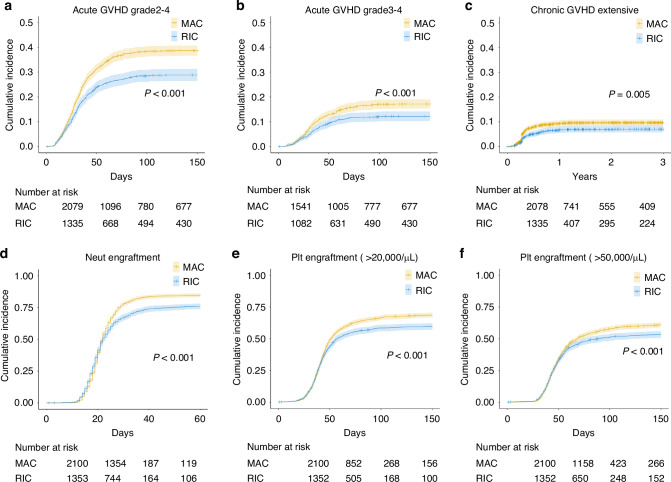
Prognostic impact of MAC vs RIC on GVHD and Engraftment. Incidence of acute GVHD **a** grade 2–4 and **b** grade 3–4, **c** chronic GVHD are shown. Engraftment defined as a sustained peripheral blood **d** neutrophil count of >500 × 10^6^/L and platelet count of **e** >20,000/μL and **f** >50,000/μL.

Then, we compared the impact of MAC and RIC on transplantation outcomes such as GVHD and engraftment. As expected, both acute GVHD and chronic GVHD were significantly more frequent in MAC than RIC. Cumulative incidence of acute GVHD grade 2–4 at 100 days was 38.4% vs 28.4% (*P* < 0.001), acute GVHD grade 3–4 at 100 days was 17.0% vs 12.1% (*P* < 0.001) and chronic GVHD at 2-year was 9.7% vs 6.9% (*P* = 0.005) (Fig. 2a–c).

Next, we assessed engraftment, and found that both neutrophil and platelet engraftment were significantly higher in MAC than RIC. The 60-day cumulative incidence of neutrophil engraftment was 84.7% vs 75.9% (*P* < 0.001) (Fig. 2d). Platelet engraftment defined as greater than 20,000/μL was 57.3% vs 51.5% (*P* < 0.001) and platelet engraftment defined as greater than 50,000/μL was 45.9% vs 42.9% (*P* < 0.001) (Fig. 2e, f).

### Multivariate analysis and subgroup analysis

In multivariate analysis for OS, RIC was related to worse outcome than MAC (HR 1.10, 95% CI: 1.01–1.21, *P* = 0.002). Other factors related to worse OS included patient age >50 years (HR 1.24, 95% CI:1.13–1.37; *P* < 0.001), male patient (HR 1.33, 95% CI: 1.18–1.49; *P* < 0.001), higher HCT-CI score (HR 1.21, 95% CI: 1.09-1.33; *P* < 0.001), higher ECOG PS (HR 1.89, 95% CI: 1.70–2.10; *P* < 0.001), non-CR state at transplantation (HR 2.13, 95% CI: 1.90–2.40; *P* < 0.001), poor chromosome risk (HR 1.87, 95% CI: 1.54–2.26; *P* < 0.001), longer time from diagnosis to CBT (HR 1.37, 95% CI: 1.20–1.56; *P* < 0.001), whereas HLA mismatch or GVHD prophylaxis did not affect OS. Notably, the CBT outcome have improved over time, with a comparison of 2016–2019 to 2010–2015 showing a reduction in OS (HR 0.83, 95% CI: 0.76–0.92; *P* < 0.001) (Table 4).

**Table 4:** Multivariate analysis of OS for all patients.

Covariates	HR	95% CI	*P* value	
Conditioning				
RIC vs MAC	1.10	1.01–1.21	0.04	*
Patient age				
≧50 vs <50	1.24	1.13–1.37	<0.001	***
Patient sex				
Male vs Female	1.33	1.18–1.49	<0.001	***
HCT-CI				
≧3 vs <3	1.21	1.09–1.33	<0.001	***
ECOG PS				
2–4 vs 0–1	1.89	1.70–2.10	<0.001	***
Disease status				
CR2 vs CR1	0.96	0.79–1.16	0.640	
Non-CR vs CR1	2.13	1.90–2.40	<0.001	***
HLA mismatch				
2 vs 0,1	0.99	0.90–1.10	0.88	
≧3 vs 0.1	0.96	0.58–1.59	0.88	
Sex mismatch				
Positive vs Negative	1.03	0.92–1.15	0.60	
GVHD Prophylaxis				
TAC used vs CSA used	0.95	0.85–1.06	0.37	
Chromosome risk				
Intermediate vs favorable	1.22	1.02–1.47	0.03	*
poor vs favorable	1.87	1.54–2.26	<0.001	***
SCT year				
2016–2019 vs 2010–2015	0.83	0.76–0.92	<0.001	***
Time from diagnosis to CBT				
3–6 months vs <3 months	1.12	0.97–1.29	0.11	
>6 months vs <3months	1.37	1.20–1.56	<0.001	***

*PS* performance status, *HCT-CI* hematopoietic cell transplantation-specific comorbidity index, *SCT* stem cell transplantation.

*p < 0.05; ***p < 0.001.

**Fig. 3:**
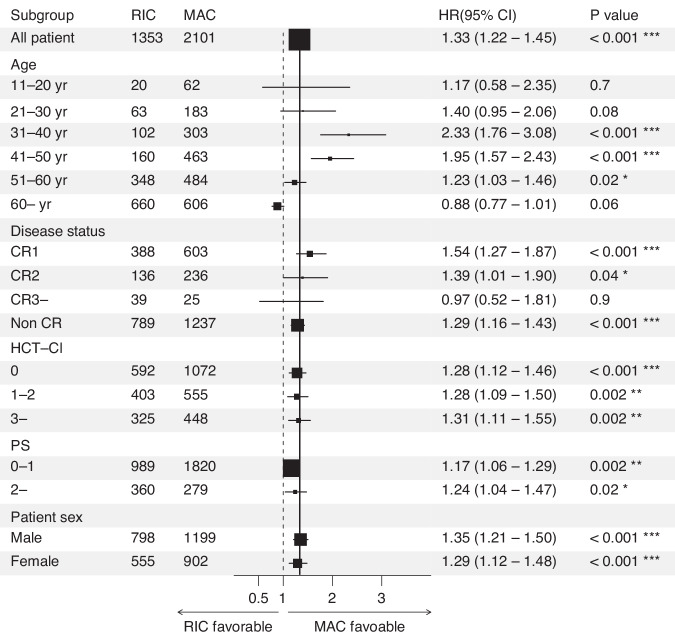
Forest plot analysis comparing MAC vs RIC. HRs with 95% CI of overall survival were calculated using univariable Cox analysis. HR of RIC vs MAC in all patients is shown on the top. The box size shows the number of patients.

To further investigate factors affecting the choice of MAC and RIC, we performed a subgroup analysis, particularly focusing on each significant factor identified in multivariate analysis. As a result, patient age at transplantation was the strongest factor influencing the outcome in RIC vs MAC. The HR for RIC was 2.33 (95% CI: 1.76–3.08, *P* < 0.001) for patients aged 31–40 and 1.95 (95% CI: 1.57–2.43, *P* < 0.001) for patients aged 41–50 indicating significantly more favorable efficacy in MAC regimens. The efficacy of MAC decreased with higher age thereafter; HR 1.23 (95%CI 1.03–1.46, *P* = 0.02) for patients aged 51–60 and 0.88 (95%CI 0,77–1.01, *P* = 0.06) for patients aged 60 or over (Fig. 3). Interestingly, other factors, except for disease status of CR3 or over, were all favorable to MAC. Taken together, these data suggest that age at transplantation is an important factor for deciding the intensity of conditioning regimens. In particular, older patients need to be carefully considered for selecting MAC.

### Detailed analyses of outcomes among middle-aged patients

**Fig. 4:**
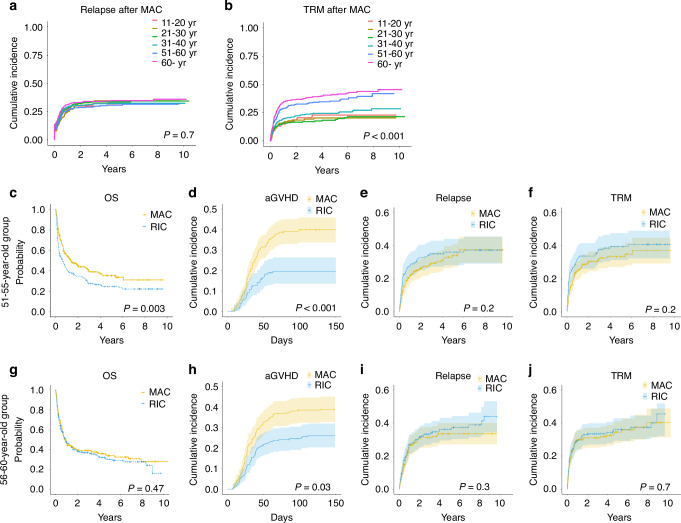
Prognostic impact of MAC vs RIC on outcomes in each age sub-group. Cumulative incidence of relapse (**a**) and treatment related mortality (**b**) in each age group are shown. Outcomes in the 51–55-year age group are shown for **c** OS, **d** acute GVHD, and cumulative incidence of **e** relapse and **f** TRM. Outcomes in the 55-60-year age group are shown for **g** OS, **h** acute GVHD, and cumulative incidence of **i** relapse and **j** TRM.

To further investigate the impact of age on selection of MAC regimens, we evaluated the cumulative incidence of relapse and TRM after MAC administration for each age group. Although the relapse rate did not differ between age groups (Fig. 4a), the TRM rate following MAC regimens increased significantly for patients in their 40 s to those in their 50 s. The 5-year cumulative incidence of TRM was 22.7% for teenagers, 20.2% for those in their 20 s, 18.2% for those in their 30 s, and 24.3% for those in their 40 s, 35.2% for those in their 50 s, and 40.9% for those over 60 (*P* < 0.001).

Then, to evaluate the safety age threshold for MAC regimens among individuals in their 50 s, where the efficacy of MAC was uncertain (Figs. 3 and 4b), we further divided 50 s into two groups: 51–55 years vs 56–60 years. For the 51–55-year group, MAC regimens showed significantly higher 5-year OS than RIC regimens (35.2% vs 24.6%, *P* = 0.003) (Fig. 4c), despite the higher incidence of acute GVHD at 100 days (39.9% vs 19.6%, *P* < 0.001) and a similar incidence of relapse and TRM (Fig. 4d–f). On the other hand, for the 56–60-year group, the superiority of MAC in terms of 5-year OS was diminished (34.0% vs 30.0%, *P* = 0.47) (Fig. 4g). The cumulative incidence of acute GVHD at 100 days was higher in MAC regimens (38.5% vs 24.6%, *P* = 0.03), although the relapse rate and TRM incidence did not differ between RIC and MAC (Fig. 4i–j).

**Fig. 5:**
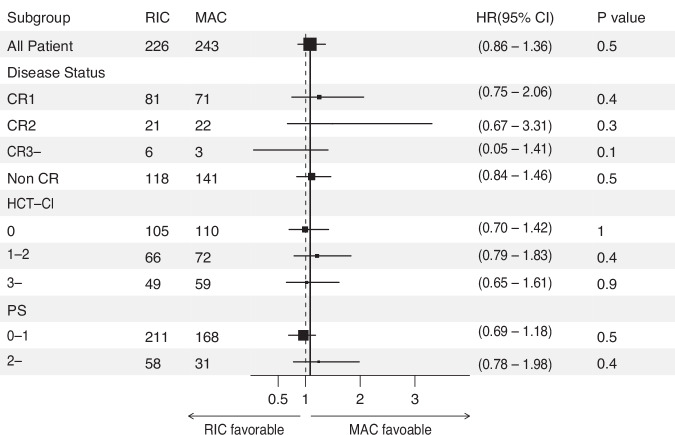
Forest plot analysis among patients aged 56–60 years comparing MAC vs RIC. HRs with the 95% CI of overall survival were calculated using univariable Cox analysis. HR of RIC vs MAC in all patients is shown on the top. The box size shows the number of patients.

Further evaluation with subgroup analysis among the 56–60-year group revealed that regardless of disease status, HCT-CI, and PS, there was no difference in OS between RIC and MAC (Fig. 5). Taken together, these results suggest RIC is suitable for patients 56 years or over, even with better PS, HCT-CI or higher disease risk, due to the similar OS with less toxicity.

## Discussion

This study represents the first large-scale, real-world data analysis comparing effects of RIC and MAC in adult AML patients undergoing CBT. Our findings indicate that age at transplantation is the most critical factor in deciding between RIC and MAC, with a threshold of 55 years. This threshold aligns with previous reports suggesting 50 or 60 years as a benchmark for AML; however, we are the first to specifically establish this threshold in the context of CBT for AML.

Although CB has known to have a potent GVL effect [31], it is widely accepted that newborn baby-derived cord blood has fewer GVL effects than adult-derived bone marrow and peripheral blood stem cells [32–34]; thus, in CBT, MAC regimens like CY/TBI (or rather intensified MAC regimens including high-dose cytarabine added on CY/TBI) are preferred in Japan [35]. From incidence curves for relapse superimposed for each age subgroup, our study indicates enhanced efficacy of MAC rather than RIC from the viewpoint of relapse suppression, regardless of patient age.

On the other hand, incidence of TRM increases non-linearly in patients in their 50s and 60s, and benefits of MAC (relapse reduction) are canceled by higher TRM. More detailed analyses indicated that MAC could be harmful for patients 56–60 years of age, even if their ECOG PS and/or HCT-CI scores are low enough, and in this cohort, RIC may be a better choice regardless of disease status, including non-CR status.

The higher incidence of TRM after MAC may be a composite outcome of various types of adverse events after CBT, including engraftment failure, infection, organ failures, and GVHD [36, 37], and in this study we focused on acute GVHD. Incidence of acute GVHD and its grade is generally higher in patients with MAC than RIC regimens, because more severe tissue damage by MAC tends to enhance severe acute GVHD more often and to a greater degree through macrophage activation via damage-associated molecular patterns (DAMPs) [38, 39]. Although increased and enhanced acute GVHD did not result in significantly worse TRM in younger patients, presumably due to a higher response rate to systemic corticosteroids compared to HCT from adult donors [40], acute GVHD could be harmful to patient quality of life or mental status [29]. Additionally, in middle aged or elderly patients, acute GVHD followed by systemic corticosteroid treatments can cause other lethal adverse events, mainly involving infections. This partially explains the significantly higher incidence of TRM in patients in their 50s or 60s. More effective prophylaxis and treatment of acute GVHD can reduce the incidence of these adverse events, and also TRM, especially in this age sub-cohort.

This study has some limitations that should be mentioned. First, some patient data in the TRUMP lacked detailed information on HLA genotype or CD34+ cell dose, so that the degree of HLA matching was based on antigen levels for HLA-A, HLA-B, and HLA-DR loci. Additionally, the analysis of CD34+ cell dose was not sufficient, as the dataset includes some outlier values, likely due to errors in digit entry during data registration. Second, we did not evaluate laboratory data, such as serum albumin or CRP [41, 42], or complex and/or monosomal karyotypes [43], which have been reported as prognostic factors for transplantation in elderly AML patients. Third, the choice of conditioning regimen intensity (MAC vs. RIC) and its detail (TBI vs non-TBI regimen) are at the discretion of the attending physician. The RIC cohort in the younger patients may have some skewed backgrounds, although no statistical differences were detected. In order to answer the question of which regimen is better, MAC versus RIC in middle-aged or elderly AML patients, a randomized clinical trial should be conducted.

In conclusion, we observed that OS, PFS, relapse rate, and engraftment rate are superior with MAC among all adult AML patients who received CBT. However, for middle-aged patients, especially those in the 56–60-year group, careful consideration is necessary when using MAC, due to the higher risk of acute GVHD without improvement in OS compared to RIC, regardless of disease status or performance status. This study is expected to serve as a standard, and with further accumulation and stratification of data, new prognostic factors are expected.

## Data Availability

The datasets generated during and/or analyzed during the current study are available from the corresponding author on reasonable request.
